# Supplementary material for: Extremely broadband, on-chip optical nonreciprocity enabled by mimicking nonlinear anti-adiabatic quantum jumps near exceptional points
Source: Nat Commun. 2017 Jan 20;8:14154. doi: 10.1038/ncomms14154 (PMC5263877; doi:10.1038/ncomms14154)
Supplement: Supplementary Information — Supplementary Figure, Supplementary Notes and Supplementary References [file ncomms14154-s1.pdf]

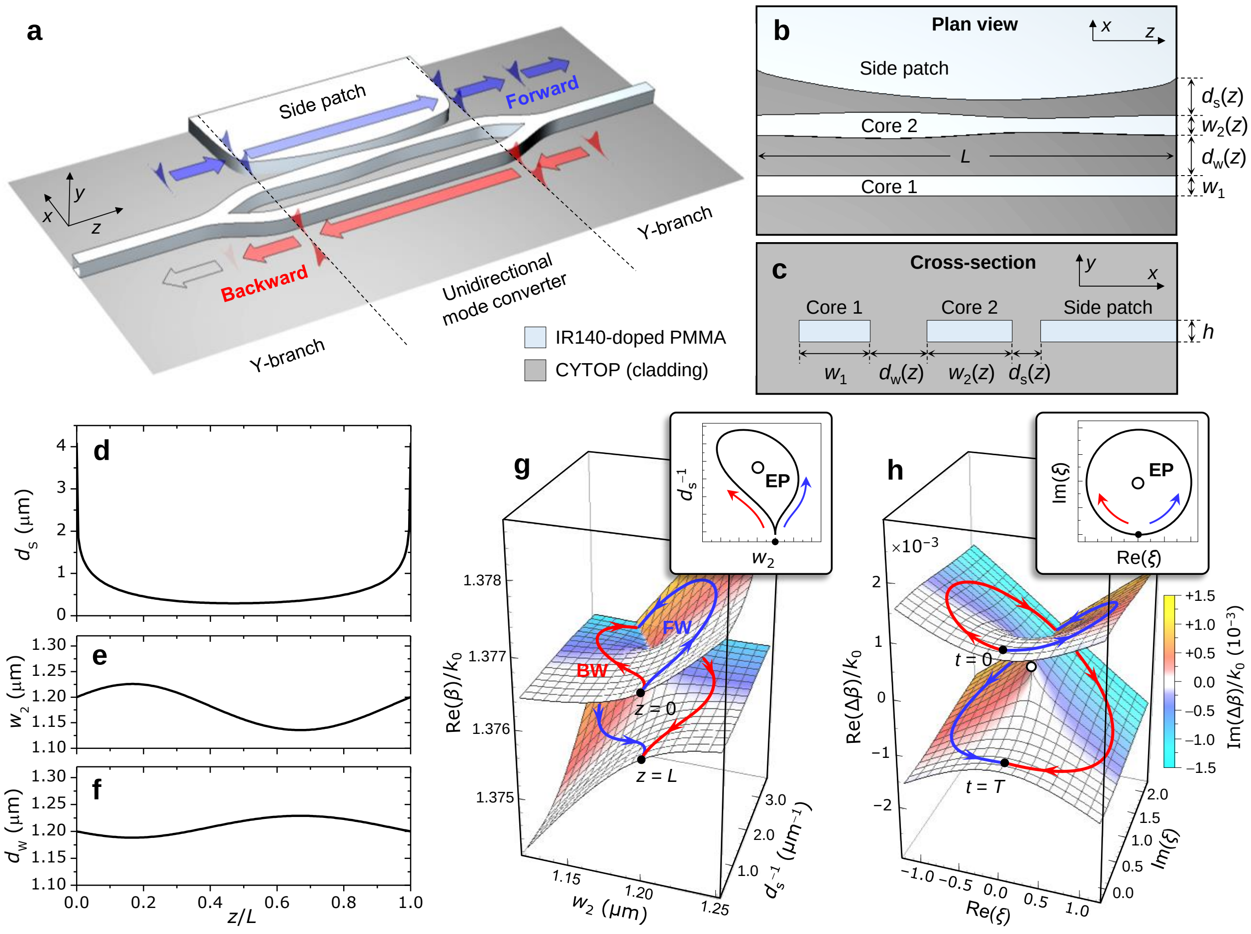

Supplementary Figure 1 | An exemplary design based on a lithographically-generated complex effective-index modulation. (**a-c**) Schematic drawing of a device design in (**a**) 3D view, (**b**) plan view in the x-z plane, and (**c**) cross-section view in the x-y plane. In this structure, the appropriately-modulated inter-waveguide spacing $d_w(z)$, waveguide width $w_2(z)$, and waveguide-side-patch spacing $d_s(z)$ create the required real and imaginary effective-index profiles. (**d-f**) Optimized $d_w(z)$, $w_2(z)$, and $d_s(z)$ profiles along the z axis for a waveguide platform based on IR140-doped PMMA core and CYTOP cladding. (**g**) Forward and backward trajectories of the instantaneous eigenvalue (normalized propagation constant, β/k_0) on a device-geometric w_2-d_s^{-1} plane (**g**) formed by the $d_w(z)$, $w_2(z)$, and $d_s(z)$ profiles in **d-f**. (**h**) Forward and backward trajectories of the instantaneous eigenvalue on a normalized complex-energy parameter Re(ξ)-Im(ξ) plane. In **g** and **h**, the inset on the top-right indicates the parametric path and location of the EP. Plot ranges along the horizontal and vertical axes are identical to those in the main plot.

Supplementary Note 1: Non-adiabatic transition in a generic time-varying non-Hermitian system

In this work, we assume a binary symmetric system where the Hamiltonian is expressed as a symmetric 2×2 matrix. For symmetric non-Hermitian Hamiltonians, a bi-orthogonal pair of eigenvector sets is given by the mutual transpose of themselves. Thereby, we use the c-product $\langle \psi^* | \psi \rangle$ that guarantees the orthonormality of the basis sets[1]. In time-varying systems, the Schrödinger-type equation for our case is written as

$$\frac{d}{dt}\left|\psi(t)\right\rangle = i\mathbf{H}(t)\left|\psi(t)\right\rangle \tag{1}$$

where $|\psi(t)\rangle$ is a dynamic state and $\mathbf{H}(t)$ is a time-dependent Hamiltonian. The dynamic state is expressed by a linear combination of the instantaneous eigenvectors as

$$\left|\psi(t)\right\rangle = \sum_{\mu} c_{\mu}(t)\left|\phi_{\mu}(t)\right\rangle, \tag{2}$$

where $|\phi_\mu(t)\rangle$ are the instantaneous eigenvectors of $\mathbf{H}(t)$ and $c_\mu(t)$ is its probability amplitude. Applying Supplementary Eq. (2) to Supplementary Eq. (1), we obtain

$$\sum_{\mu}\left\{\dot{c}_{\mu}(t)\left|\phi_{\mu}(t)\right\rangle + c_{\mu}(t)\left|\dot{\phi}_{\mu}(t)\right\rangle - ic_{\mu}(t)\lambda_{\mu}(t)\left|\phi_{\mu}(t)\right\rangle\right\} = 0 \ . \tag{3}$$

Applying the c-product projection of Supplementary Eq. (3) onto $\langle \phi_\nu^* |$, we obtain

$$\sum_{\mu}\left\{\dot{c}_{\mu}(t)\delta_{\nu\mu} + c_{\mu}(t)\left\langle\phi_{\nu}^{*}(t)\middle|\dot{\phi}_{\mu}(t)\right\rangle - ic_{\mu}(t)\lambda_{\mu}(t)\delta_{\nu\mu}\right\} = 0 \ . \tag{4}$$

Supplementary Eq. (4) immediately implies

$$\dot{c}_{\nu}(t) = i\lambda_{\nu}(t)c_{\nu}(t) - \sum_{\mu} g_{\nu\mu}c_{\mu}(t) = 0 \ , \tag{5}$$

where $g_{\nu\mu}(t) \equiv \langle \phi_\nu^*(t) | \partial_t \phi_\mu(t) \rangle$ denotes the instantaneous non-adiabatic coupling amplitude. This amplitude is zero for $\mu = \nu$ by virtue of the following equation,

$$\frac{d}{dt}\left\langle\phi_{\mu}^{*}(t)\middle|\phi_{\mu}(t)\right\rangle = \left\langle\dot{\phi}_{\mu}^{*}(t)\middle|\phi_{\mu}(t)\right\rangle + \left\langle\phi_{\mu}^{*}(t)\middle|\dot{\phi}_{\mu}(t)\right\rangle = 2\left\langle\phi_{\mu}^{*}(t)\middle|\dot{\phi}_{\mu}(t)\right\rangle = 0 \tag{6}$$

The non-adiabatic coupling amplitude for $\mu \neq \nu$ is expressed in terms of a time-derivative of the Hamiltonian. Differentiating the instantaneous eigenvalue equation yields

$$\dot{H}(t)\left|\phi_{\mu}(t)\right\rangle + H(t)\left|\dot{\phi}_{\mu}(t)\right\rangle = \dot{\lambda}_{\mu}(t)\left|\phi_{\mu}(t)\right\rangle + \lambda_{\mu}(t)\left|\dot{\phi}_{\mu}(t)\right\rangle \ , \tag{7}$$

and taking the c-product with $\langle \phi_\nu^* |$, we get

$$\left\langle\phi_{\nu}^{*}(t)\middle|\dot{H}(t)\middle|\phi_{\mu}(t)\right\rangle + \left\langle\phi_{\nu}^{*}(t)\middle|H(t)\middle|\dot{\phi}_{\mu}(t)\right\rangle = \left\langle\phi_{\nu}^{*}(t)\middle|\dot{\lambda}_{\mu}(t)\middle|\phi_{\mu}(t)\right\rangle + \left\langle\phi_{\nu}^{*}(t)\middle|\lambda_{\mu}(t)\middle|\dot{\phi}_{\mu}(t)\right\rangle \ . \tag{8}$$

Solving Supplementary Eq. (8) for the c-product $\langle \phi_\nu^*(t) | \phi_\mu(t) \rangle$ yields a closed-form expression for the non-adiabatic coupling amplitude such that

$$g_{\mu\nu}(t) = \left\langle\phi_{\nu}^{*}(t)\middle|\dot{\phi}_{\mu}(t)\right\rangle = \frac{\left\langle\phi_{\nu}^{*}(t)\middle|\dot{H}(t)\middle|\phi_{\mu}(t)\right\rangle}{\lambda_{\mu} - \lambda_{\nu}} \ . \tag{9}$$

Now, we write Supplementary Eq. (5) in matrix form:

$$\frac{d}{dt}\begin{pmatrix} c_\mu(t) \\ c_\nu(t) \end{pmatrix} = i\begin{pmatrix} \lambda_\mu(t) & -ig_{\mu\nu}(t) \\ ig_{\mu\nu}(t) & \lambda_\nu(t) \end{pmatrix}\begin{pmatrix} c_\mu(t) \\ c_\nu(t) \end{pmatrix}. \quad (10)$$

When an initial state is given by one of the instantaneous eigenvectors, $c_\mu(0) = 1$ and $c_\nu(0) = 0$, the non-adiabatic transition from $|\phi_\mu\rangle$ to $|\phi_\nu\rangle$ is approximately determined by the following relation

$$C_{\nu\mu}(t) = \frac{c_\nu(t)}{c_\mu(t)} = \frac{\langle \phi_\nu^* | \psi \rangle}{\langle \phi_\mu^* | \psi \rangle} \approx \int_0^t g_{\nu\mu}(t')\exp\left\{-i\int_{t'}^{t}\left[\lambda_\nu(t'') - \lambda_\mu(t'')dt''\right]\right\}dt' \quad (11)$$

in the limit that $|c_\nu(t)| \ll |c_\mu(t)|$. This relation is identical to Eq. (4) in the main text. In non-Hermitian cases where the eigenvalues include significant imaginary parts, Supplementary Eq. (11) implies that the standard quantum adiabatic theorem fails to properly describe the state evolution in the following two aspects. First, the adiabatic condition for the dynamic state $|\psi(t)\rangle$ to follow the instantaneous eigenvector $|\phi_\mu(t)\rangle$ is significantly relaxed for $|\phi_\mu(0)\rangle = |\phi_G(0)\rangle$ and $\mathrm{Im}(\Lambda_\nu - \Lambda_\mu) = \mathrm{Im}(\Lambda_L - \Lambda_G) > 0$. In Supplementary Eq. (11) (Eq. (4) in the main text), a contribution of an instantaneous coupling amplitude $g_{LG}(t')dt'$ for the infinitesimal time interval dt' to $C_{LG}(t)$ decays exponentially with the factor $\exp[-\mathrm{Im}(\Lambda_L - \Lambda_G)(t-t')]$. Therefore, $|\psi(t)\rangle$ follows the instantaneous eigenvector $|\phi_G(t)\rangle$ for $\mathbf{H}(t)$ varying quickly in time even beyond the standard quantum-adiabatic condition. This type of super-adiabatic evolution is expected for evolution paths along $\lambda_G(t)$ and its time reversal $\lambda_G(T-t)$. In another aspect, for the case $|\phi_\mu(0)\rangle = |\phi_L(0)\rangle$ and $\mathrm{Im}(\Lambda_\nu - \Lambda_\mu) = \mathrm{Im}(\Lambda_G - \Lambda_L) < 0$, corresponding to an initial evolution path along $\lambda_L(t)$ and its time reversal $\lambda_L(T-t)$ in Fig. 2b in the main text, the failure of the standard quantum adiabatic theorem is much more radical, as an anti-adiabatic state jump occurs regardless of how slowly the Hamiltonian $\mathbf{H}(t)$ changes in time. In this case, a contribution of the instantaneous coupling amplitude $g_{GL}(t')dt'$ to $C_{GL}(t)$ grows exponentially with a factor $\exp[+\mathrm{Im}(\Lambda_L - \Lambda_G)(t-t')]$. Therefore, $|\psi(t)\rangle$ undergoes a drastic transition from $|\phi_L\rangle$ to $|\phi_G\rangle$ when enough evolution time T has occurred for $T \geq T_c \equiv \mathrm{Im}(\Lambda_L - \Lambda_G)^{-1}$. This implies that $|\phi_L\rangle$ can be occupied only within a transient time period $\sim T_c$.

Supplementary Note 2: A lithographic approach for complex effective-index modulation

Confirming feasibility of the proposed lithographic approach illustrated in Supplementary Fig. 1a-c, and identically in Fig. 7 in the main text, we theoretically treat a polymer waveguide architecture based on 400-nm-thick dye-doped poly(methyl-methacrylate) (PMMA) waveguides ($n = 1.48$) embedded in low-index CYTOP cladding ($n = 1.34$). IR140 dye is uniformly doped in PMMA waveguides as an optical gain agent for operation over its gain bandwidth (~ 60 nm) spanning a wavelength range from 850 nm to 910 nm.[2] We carefully design the inter-core spacing $d_w(z)$, core-2 width $w_2(z)$, and core-to-side-patch gap width $d_s(z)$ profiles such that they precisely create the required complex effective-index profiles of the configuration indicated in Fig. 1b in the main text. For the sake of simplicity in potential fabrication, a gauge transformation scheme is applied such that the complex effective-index modulation exists only in the core-2 waveguide while maintaining parametric properties of the effective Hamiltonian including the desired EP, encircling-an-EP evolution path, and significant imaginary-eigenvalue splitting in the non-Hermitian domain. This is done by keeping the difference in the effective index modulation profiles between the two waveguides identical to that for the configuration in the previous sections where the antisymmetric effective index modulations apply to the two waveguide cores simultaneously.

Parametric optimization under this approach requires careful evaluation of the mode's complex effective indices in the core 1 and 2 waveguides, inter-waveguide coupling strength, and the consequent eigensystem properties

depending on the d_w, w_2, and d_s values within certain parametric ranges favorable for fabrication. We use the full-vectorial 3D finite-element method (Comsol Multiphysics^{TM}) for the numerical optimization. The optimized $d_w(z)$, $w_2(z)$, and $d_s(z)$ profiles are shown in Supplementary Figs. 1d-f. These profiles result in the gauge-transformed effective index modulation profiles of a configuration dictated by Eqs. (1) and (2) in the main text, where we specify $\Delta n_0 = 5.0\times10^{-4}$ at the IR140-dye's fluorescence peak wavelength of 880 nm. Notably, the obtained $d_w(z)$, $w_2(z)$, and $d_w(z)$ profiles including a waveguide-width modulation amplitude of 90 nm and a minimal critical dimension of 294 nm can be readily created using standard electron-beam lithography capable of defining a minimum feature of ~5 nm and a beam-positioning accuracy of ~0.5 nm on conventional wafer-scale areas. Resultant eigenvalue surfaces and the instantaneous eigenvalue (normalized propagation constant β/k_0 of the coupled-waveguide eigen modes) trajectories on a device-parametric w_2-d_s plane is shown in Supplementary Fig. 1g. The eigenvalue surfaces and trajectories therein clearly confirm that this example design generates the desired encircling-an-EP parametric evolution with essential ingredients such as a binary non-Hermitian eigensystem with a strong imaginary-eigenvalue ($\mathrm{Im}(\Delta\beta)/k_0$) splitting property. In particular, a formal mapping of Supplementary Fig. 1g onto a Hamiltonian-parametric $\mathrm{Re}(\xi)$-$\mathrm{Im}(\xi)$ plane is exactly identical to that of Fig. 2b in the main text as shown in Supplementary Fig. 1h. Note an exactly circular parametric path and the geometric structures of the eigenvalue-difference ($\Delta\beta/k_0$) spectra. Therefore, the essential requirements for our nonreciprocal device concept can be efficiently generated by lithography (with the additional gauge transformation scheme for experimental convenience).

Applying the full-vectorial alternating-direction-implicit 3D finite-difference beam-propagation method[3] for nonlinear modelling to this design, with the additional specifications of an IR140-doping concentration of 0.8 wt% (5.2×10^{18} cm^{-3}) that yields an optical gain constant value of 680 dB·cm^{-1} under an optical pumping density 43.4 mJ·cm^{-2} at 810 nm following the experimental data used in [2], we obtain a nonreciprocal transmission ratio (NTR) value of 4.08 dB for a device length L = 1 mm. Although we are not capable of modelling longer devices with the computational resources presently available, we note that this result implies a higher NTR value of 18.1 dB for a 5-mm-long device following the T^2-rule in Eq. (5) in the main text. Importantly, this NTR value is fairly consistent with the NTR values in Fig. 6a in the main text for a direct index-modulated device at a wavelength of 1.0 μm at which the parametric evolution path with respect to the EP is identical. Hence, a similar broadband property is expected in this specific design.

Further discussing the operating bandwidth, a limiting factor is the optical gain bandwidth of the emitter used as the optical gain agent. The gain bandwidth of conventional fluorescent dyes is on the order of 50 nm. In our case, IR140-doped PMMA has a full-width at half-maximum emission bandwidth of 60 nm.[2] Therefore, given that the nonreciprocal bandwidth of the proposed concept is greater than several 100 nm, the overall operating bandwidth using fluorescent dyes should be limited only by the dye molecule's gain bandwidth.

In an additional consideration, a Kramers-Kronig relation linking the real and imaginary dielectric constant spectra is of interest because it may reveal significant effective index perturbations at different operating wavelengths. In our case, the gain spectrum of IR140-doped PMMA takes on a Lorentzian form for the imaginary dielectric constant $\varepsilon''(\nu) = -G_0\gamma^2[(\nu-\nu_0)^2+\gamma^2]^{-1}$, where G_0, ν_0, and γ denote the peak gain factor, the peak frequency, and the half-width at half-maximum bandwidth, respectively. The Kramers-Kronig relation results in a spectral change of the real dielectric constant following an expression $\Delta\varepsilon'(\nu) = -G_0\gamma(\nu-\nu_0)[(\nu-\nu_0)^2+\gamma^2]^{-1}$. Note that the spectral profiles of $\varepsilon''(\nu)$ and $\Delta\varepsilon'(\nu)$ have opposite symmetries with respect to ν_0, potentially resulting in spectrally uneven shifts of the real and imaginary dielectric constants. In a device using position-dependent doping, the peak gain factor G_0 becomes a function of z as determined by the dye doping concentration. Consequently, the spectral real-imaginary index relation produces a spatially uneven $\Delta\varepsilon'$, which makes it difficult to create precise complex

effective index modulation profiles that persist over the spectral region of interest. However, in the proposed lithographic modal effective-index modulation approach, this problem is insignificant or might be fully neglected due to two main reasons: First, G_0 is constant over the whole device region. Second, although the complex modal effective index values change with $\varepsilon''(\nu)$ and $\Delta\varepsilon'(\nu)$ (even in the lithographic approach), the difference in the complex effective-index modulation profiles between the two coupled waveguides is unaffected, as far as the wavefunctions of the guided modes are not significantly modified under $G_0 << |\varepsilon_{\text{host}}|$, where $\varepsilon_{\text{host}}$ denotes the dielectric constant of the host medium, i.e., PMMA in our case. In our example design in Supplementary Fig. 1, $G_0 = 5\times10^{-4} << |\varepsilon_{\text{host}}| = 2.19$ (a similar condition generally applies to other species of dye molecules and host materials). As we previously explained in association with the index-difference-keeping gauge transformation, the essential evolution dynamics that we demand is precisely created as long as the difference in the complex effective-index modulation profiles between the two coupled waveguides is maintained.

Supplementary References

[1] Moiseyev, N. Quantum theory of resonances : calculating energies, widths and cross-sections by complex scaling. *Phys. Rep*. **302**, 211–293 (1998).

[2] Keshmarzi, E. F., Tait, R. N., & Berini, P. Near infrared amplified spontaneous emission in a dye-doped polymeric waveguide for active plasmonic applications. *Opt. Express* **22**, 12452–12460 (2014).

[3] Shibayama, J., Yokomizo, A., Yamauchi, J., and Nakano, H. Simplified algorithms for the full-vectorial ADI-BPM using a fundamental scheme. *IEEE Photon. Technol. Lett.* **25**, 147150 (2013).
